# Characterization of Aroma-Active Components and Antioxidant Activity Analysis of E-jiao (*Colla Corii Asini*) from Different Geographical Origins

**DOI:** 10.1007/s13659-017-0149-3

**Published:** 2018-02-27

**Authors:** Shan Zhang, Lu Xu, Yang-Xi Liu, Hai-Yan Fu, Zuo-Bing Xiao, Yuan-Bin She

**Affiliations:** 1 State Key Laboratory Breeding Base of Green Chemistry-Synthesis Technology, College of Chemical Engineering, Zhejiang University of Technology, Hangzhou, 310014 China; 2 College of Material and Chemical Engineering, Tongren University, Tongren, 554300 Guizhou China; 3 School of Pharmaceutical Sciences, South-Central University for Nationalities, Wuhan, 430074 China; 4 School of Perfume and Aroma Technology, Shanghai Institute of Technology, Shanghai, 201418 China

**Keywords:** *Colla Corii Asini*, Aroma-active compounds, Antioxidant activity, Chemometrics

## Abstract

**Abstract:**

E-jiao (*Colla Corii Asini*, CCA) has been widely used as a healthy food and Chinese medicine. Although authentic CCA is characterized by its typical sweet and neutral fragrance, its aroma components have been rarely investigated. This work investigated the aroma-active components and antioxidant activity of 19 CCAs from different geographical origins. CCA extracts obtained by simultaneous distillation and extraction were analyzed by gas chromatography–mass spectrometry (GC–MS), gas chromatography–olfactometry (GC–O) and sensory analysis. The antioxidant activity of CCAs was determined by ABTS and DPPH assays. A total of 65 volatile compounds were identified and quantified by GC–MS and 23 aroma-active compounds were identified by GC–O and aroma extract dilution analysis. The most powerful aroma-active compounds were identified based on the flavor dilution factor and their contents were compared among the 19 CCAs. Principal component analysis of the 23 aroma-active components showed 3 significant clusters. Canonical correlation analysis between antioxidant assays and the 23 aroma-active compounds indicates strong correlation (r = 0.9776, p = 0.0281). Analysis of aroma-active components shows potential for quality evaluation and discrimination of CCAs from different geographical origins.

**Graphical Abstract:**

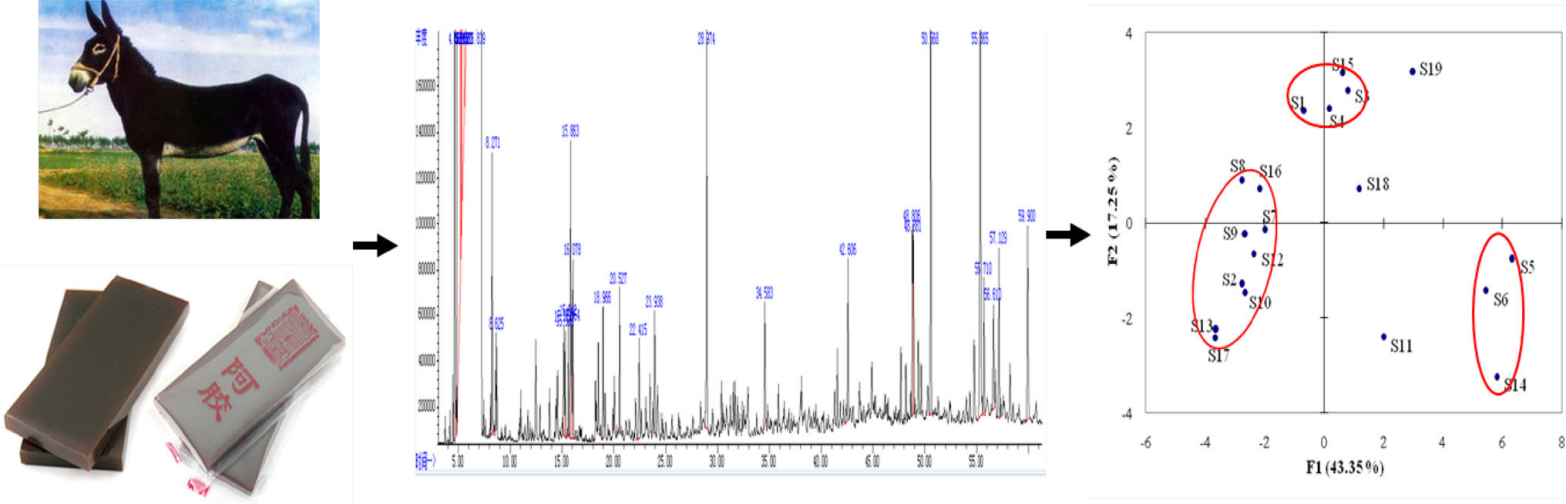

## Introduction

E-jiao (*Colla Corii Asini*, CCA), made of donkey-hide gelatin, rice wine, rock candy and soya bean oil, has been widely used as a functional food and an ingredient in traditional Chinese medicine (TCM) [1–3]. Together with ginseng and pilose antler, CCA has been recognized as one of the “Three Treasures for Nourishing” since ancient times [4]. In TCM, CCA has been mainly used as a clinical hematic antanemic therapy and to treat a variety of conditions, including bleeding, dizziness, insomnia and a dry cough, for more than 2000 years [5]. CCA has demonstrated various biological activities in modern pharmacological investigations, including hemostasis, anti-aging activity, anti-tumor, immunomodulatory, bone repairing, anti-inflammatory, anti-fatigue, etc. [6].

The quality and chemical composition of CCA depend on many factors, such as geographical origins, raw materials, water sources, temperature and processing. Among various factors, geographical origin is highly correlated with other factors and is known to confer specific healthy and nutritional qualities to CCA. Traditionally, CCA is named and evaluated by its place of origin and quality difference exists among CCAs from different producing areas, e.g., for a long time Dong-E county of Shandong province has been recognized as the origin of authentic and top-quality CCA products [7]. Therefore, it is necessary to develop reliable and effective analytical methods for quality evaluation of CCAs from different geographical origins.

As a complex mixture, various components have been separated and identified from CCA, including collagen, amino acids, proteins, polysaccharides, volatile substances, trace elements, etc. [8, 9]. Authentic CCA is characterized by its typical sweet and neutral fragrance, which is one of the most significant factors that shape the quality and affect consumer behaviors. However, most of the current investigations on CCA have been devoted to the analysis and bioactive effects of the non-volatile substances [10, 11] and the investigations on volatile components of CCA have been rarely reported. A series of methods can be used to obtain the volatile flavour compounds of CCA like distillation [12], Headspace-solid phase microextraction (HS-SPME) [13], solid-phase extract (SPE) [14], simultaneous distillation and extraction (SDE) [15, 16] and so on. Mao et al. [17] studied the volatile components of CCA from Shangdong by gas chromatography–mass spectrometry (GC–MS) analysis of distilled samples. They identified 23 volatile ingredients and 12 of them have characteristic smells. However, the contributions of the specific components to CCA aroma and the composition variations of volatiles in CCAs from different producing areas were not investigated. Although CCA contains many volatile components, only a small fraction of them contributes to its overall aroma. Therefore, besides qualitative and quantitative analysis of volatile components, some complementary sensory techniques, such as gas chromatography–olfactometry (GC–O), are required to evaluate the aroma contributions of volatile components. Aroma extract dilution analyses (AEDA) is a frequently used method combined with GC–O, which has been used to identify the key aroma compounds in foods [18], beverage like mushroom [19, 20], durian wine [21] and so on.

Recently, CCA has drawn continuously increasing attention due to its potential anti-aging and antioxidant effects. In 2012, Wang et al. [22] investigated the potential anti-aging effect of CCA using D-galactose (gal) induced aged model mice. Their results indicated that CCA could improve the antioxidant activities of superoxide dismutase (SOD), catalase (CAT) and glutathione peroxidase (GSH-Px), scavenge free radicals such as malondialdehyde (MDA), as well as modulate the expression of aged-related gene. Such investigations have proved the anti-aging and antioxidant effects of CCA and cast new light on its anti-aging mechanisms. The general antioxidant activity analysis will bring an important criterion for quality evaluation of different CCA samples. However, to our knowledge, little information about the general in vitro antioxidant activity of CCA has been reported to enable quality evaluation and comparison among CCAs from different geographical origins. Therefore, the aim of the present study was: (1) to identify, quantify and attribute the aroma contributions of the volatile compounds in CCA using GC–MS/GC–O–AEDA techniques and (2) to evaluate the quality of CCAs from different producing areas by analysis of antioxidant activity and key aroma profile using chemometrics.

## Materials and Methods

### Collection of CCA Samples and Chemicals

Nineteen CCAs from different geographical origins were collected from the market branch of each producer. All the samples were stored in a cool, dark area before analysis of aroma components and antioxidant activities. Information concerning the geographical origins and brands of the 19 CCA samples is listed in Table 1.

**Table 1:** Ninteen kinds of CCA samples

CCA sample code	Brand	Produsing area: province
S1	Dong-E	ShanDong
S2	Fu Jiao	ShanDong
S3	Shi Meisheng	ShanDong
S4	Dong Teng	ShanDong
S5	Sheng Liyuan	ShanDong
S6	Hua Xin	ShanDong
S7	Shou Kang	ShanDong
S8	Pu Yang	HeNan
S9	Wei Xin	HeNan
S10	Fu Ren	HeNan
S11	Si Fang	HeNan
S12	Dong Jian	HuNan
S13	Yong Jia	HuNan
S14	Kang Yuan	HuBei
S15	Hua Guang	HuBei
S16	Hua Long	HeBei
S17	San Jiao	XinJiang
S18	Tong Rentang	BeiJing
S19	Zhang Jiachuan	GanSu

1,1-Diphenyl-2-picrylhydrazyl (DPPH) was supplied by Sigma-Aldrich (Steinheim, Germany). 2,2-azino-bis(3-ethylbenzothiazoline-6-sulphonic acid) diammonium salt (ABTS) was purchased from the Beyotime Institute of biotechnology (Nantong, China). 1-Decanol and standard aroma compounds were obtained from Sigma-Aldrich (Shanghai, China) and Apple Flavor and Fragrance Co. Ltd. (Shanghai, China). The other chemicals and solvents used in this study came from Sinopharm Chemical Reagent Co. Ltd. (Shanghai, China). All of them were analytical reagents.

### Extraction of the Volatile Compounds

The simultaneous distillation extraction (SDE) technique was used to obtain aromatic extracts of CCA. Twenty grams of CCA and 100 mL pure water were put into a 500 mL distillation flask which was placed in a heater fixed at 80 °C. Dichloromethane (60 mL) was pipetted into a 100 mL distilled flask, which was placed in a water bath at 62 °C. SDE was performed for 3 h. After extraction, 100 μL of internal standard (1-decanol) was added to the distillate. The extract was dehydrated by anhydrous sodiumsulfate, and then condensed to 200 μL under a gentle stream of pure nitrogen.

### GC–MS, GC–Flame Photometric Detector (FPD) and GC–O analysis

The GC–MS analysis was performed using a Hewlett-Packard 7890A GC with a 5975C mass selective detector (MSD) (Agilent Technologies, USA) working under electron ionisation (EI) circumstance (70 eV, ion source temperature 230 °C) with the ion trap operating in a scanning mode (the scan range was 30–450 m/z at a scan rate of 1 scan/s). The separation was performed using DB-INNOWAX column (60 m × 0.25 mm i.d. × 0.25 μm film thickness, Agilent Technologies, USA) and DB-5 analytical fused silica capillary column (60 m × 0.25 mm i.d. × 0.25 μm film thickness, Agilent Technologies, USA). Helium (purity 99.999%) was used as the carrier gas with a constant flow velocity of 1 mL/min. The temperature of quadrupole mass filter was set at 150 °C. The transfer line temperature was set at 250 °C. The oven temperature was 60 °C ramped at the rate of 3 °C/min to 230 °C and held for 10 min. The volatile compounds were positively identified by comparing retention indices (RIs) and retention times with those obtained for authentic standards, or by matching the measured mass spectra with the standards in the Wiley 7n.l Database (Hewlett-Packard, Palo Alto, CA). The RIs were determined via sample injection with a homologous series of alkanes (C6–C30) (Sigma-Aldrich, St. Louis, MO, USA). GC–O was performed on a 7890A GC equipped with a flame ionization detector (FID), olfactory detection port (ODP, Gerstel, Mülheiman der Ruhr, Germany). Working conditions were same to the GC–MS. Postcolumn flow was split at a ratio of 1:1 to the FID and the ODP using two deactivated and uncoated fused silica capillaries (106 cm × 0.15 mm I.D., 139 cm × 0.1 mm I.D.) at the end of the capillary. Each sample was run in triplicate.

Sulfur compounds were detected using a flame photometric detector (FPD) (Agilent Technologies, USA) installed on the Agilent 7890A GC. Separations were accomplished using two different capillary columns as described above in the GC–MS and GC–O analysis. The oven temperature program used was the same as that employed for the GC–O studies. Identification of sulfur compounds was based on matching RIs from authentic sulfur standards and RIs reported in the literature with those observed in the samples.

The quantification was performed using the internal standard method. A set of standard mixtures, previously prepared and containing known concentrations of the chemical standards ($C_{analyte}$) and the I.S. concentration ($C_{I.S.}$), were analyzed and their peak areas ($A_{analyte}$ and $A_{I.S.}$) recorded. For each chemical standard, a six-point calibration line of relative peak area ($A_{analyte} /A_{I.S.}$) versus $C_{analyte}$ was drawn to confirm a linear detector response, from which the amount of the analyte could be determined. The unknown concentration of an analyte ($C_{analyte,X}$) was calculated according to the interpolation of the calibration line as:

$$C_{analyte,X} = \left( {A_{analyte} /A_{I.S.} } \right) \times \left( {C_{I.S.} /DRF_{analyte} } \right)$$

where $DRF_{analyte}$ was the Detector Response Factor (DRF) for an individual analyte. The internal standard (1-decanol) using a DRF of 1.0.

### Aroma Extract Dilution Analysis (AEDA)

The original aroma extracts were also analyzed by GC–O to evaluate the contributions of the identified volatile components to CCA odor. For AEDA, the concentrated aromatic extract (200 μL) of CCA was 1:2 diluted stepwise using dichloromethane as the solvent to obtain serial dilutions (1:2, 1:4, 1:8 and up to 1:64) of the original extracts. Sniffing of dilutions was continued until no odorant could be detected by GC–MS and GC–O. Each odorant was thus assigned a flavour dilution (FD) factor representing the last dilution in which the odorant was still detectable. The larger the FD factor of an aroma component, the higher the degree of its flavour activity [23].

### Sensory Assessment of CCA

Sensory analysis of CCA samples was performed by a group of 12 members, including six females and six males between 22 and 40 years old. A CCA sample (2.00 g) was dissolved in pure water (10 mL) and then placed in a tasting opaque glass coded for a specific number with three digits. The temperature of samples was kept at 50 ± 2 °C during sensory analysis. This assessment consisted of 8 aroma descriptors (toasted, muttony, medicinal, fatty, milky, earthy, sour and waxy). The intensity from each panelist was given a score of 0–9 where 0 = none or not perceptible intensity, and 9 = extremely high intensity. The intensity of samples was normalized to reduce the response differences among the panelists. Results were expressed as the median intensity of the sensory perceptions of the 12 panelists. All of the samples were evaluated in triplicate.

### ABTS Radical Cation Scavenging Activity

The determination of ABTS radical scavenging was carried out as reported by Luo et al. [24] with slight modifications. The stock solutions included ABTS solution and oxidant solution. The working solution was prepared by mixing the two stock solutions in equal quantities and allowing them to react for 16 h at room temperature in the dark. The solution was then diluted by mixing 1 mL working solution with 90 mL ethanol (80%) in order to obtain an absorbance of 0.7 ± 0.05 at 734 nm. A fresh ABTS solution was prepared for each assay. Samples (10 μL) with a concentration range of 2.00–20.00 mg/mL were mixed with 200 μL of fresh ABTS solution and the mixture was left at room temperature for 6 min. The absorbance was then measured at 734 nm.

The ABTS radical scavenging activity of an unknown sample was calculated as follows:

$${\text{ABTS}}\left( {\text{\% }} \right) = \left[ {1 - \left( {A_{1} - A_{2} } \right)/A_{3} } \right] \times 100$$

where $A_{1}$ is the absorbance in the presence of the test sample; $A_{2}$ is the absorbance of contrast and $A_{3}$ is the absorbance of the control (ABTS solution without test sample). The values of the half maximal inhibitory concentration (IC_50_) were determined as reported above. Tests were carried out in triplicate.

### DPPH Radical Cation Scavenging Activity

DPPH radical scavenging activity was determined using the method as described by Brand-Williams and coworkers [25] with slight modifications. Samples (100 μL) with a concentration range of 2.00–50.00 mg/mL were added to 100 μL DPPH solution. The mixture was stirred vigorously and allowed to stand at room temperature in the dark for 30 min. The absorbance of the resulting solution was measured at 517 nm. A lower absorbance of the reaction mixture indicates higher free radical scavenging activity, and vice versa for higher absorbance. Antioxidant activities of test compounds were expressed as IC_50_, which is defined as the concentration of the test material required to cause a 50% decrease in initial DPPH concentration.

The DPPH radical scavenging activity of each sample was calculated as the percent inhibition according to the following equation:

$${\text{DPPH}}\left( {\text{\% }} \right) = \left[ {1 - \left( {A_{1} - A_{2} } \right)/A_{3} } \right] \times 100$$

where $A_{1}$ is the absorbance in the presence of the test sample; $A_{2}$ is the absorbance of contrast and $A_{3}$ is the absorbance of the control (DPPH solution without test sample). Tests were carried out in triplicate.

### Chemometrics

Principal components analysis (PCA) of the aroma-active components was performed on XLStat 2010 (Addinsoft, New York, USA) to demonstrate the distribution and clustering of the 19 CCAs by dimension reduction. Canonical correlation analysis (CCA) was performed on Matlab 7.0.1 (Mathworks, New Mexcico, USA) to investigate the correlation between antioxidant assay values (ABTS and DPPH) and the aroma-active components. The calibration of ABTS and DPPH values was performed using the routines in the SPSS 19.0 software (SPSS Inc., Chicago, USA).

## Results and Discussion

### GC–O Dilution Results

Volatile components with high concentrations do not always play important roles in odor contribution, because the odor level of a volatile component was also linked with its threshold. GC–O can easily differentiate which compounds have nothing to do with odor and which are important aroma-active components although they were present at trace amount [26, 27].

The Dong-E CCA was used as the typical sample to define the common aroma-active volatiles in the 19 CCAs. A total of 23 aroma-active compounds were identified by GC–O and AEDA as shown in Table 2. Three compounds were found to be the most potent aroma-active compounds among the 23 identified components, including s-methyl thioacetate, 2,6-dimethylpyrazine and 2-ethyl-3,6-dimethylpyrazine. They were detected as having the highest FD factors. The odour characteristic of s-methyl thioacetate was reported to be sulfurous and burnt. It is a well-known powerful odorant that contributes to the characteristic aromas of various foods like onion, garlic and radish, due to its low odor threshold. Compounds 2,6-dimethylpyrazine and 2-ethyl-3,6-dimethylpyrazine exhibited “roasted nut”, and “roast” notes, respectively. Their highest FD factor values suggested they are likely to make the main contributions to the toasted aroma of CCA. Pyrazines are mostly formed through the Maillard reaction between saccharides and amino residues [28, 29]. A higher temperature would benefit the Maillard reaction, and produce more pyrazines. Similar to pyrazines, a higher temperature also facilitates furan formation through nonenzymic browning of sugars. Several furans were identified in this study, such as 2-pentyl-furan and 5-methyl furfural. The presence and the contents of various pyrazines and furans can reflect the operation parameters of heating process during the manufacturing of CCA.

**Table 2:** Odor-active Volatiles in Dong-E CCA

No.	RI		Aroma compound	Odor description	FD factor	Identification^a^
	INNOWAX	DB-5				
1	716	505	Dimethyl sulfided	Cooked onion, sulfur, gasoline	16	RI, O, S
2	767	754	s-ethyl thioacetate	Sulfurous, fruity	8	RI, O, S
3	1061	794	Dimethyl disulfide	Cabbage, putrid	32	RI, O, S
4	1075	701	s-methyl thioacetate	Sulfurous, burnt	> 64	RI, O, S
5	1097	802	hexanal	Apple, cut grass	32	RI, O, S
6	1098	821	3-Methyl-2-butene-1-thiol	Amine, smoke	8	RI, O, S
7	1240	991	2-Pentyl-Furan	Green, leafy	2	RI, O, S
8	1328	905	2,5-Dimethylpyrazine	Cocoa, medicine	16	RI, O, S
9	1330	885	2,6-Dimethylpyrazine	Roasted nut, cocoa	> 64	RI, O, S
10	1341	976	2-Ethyl-5-methylpyrazine	Nutty, roasted	8	RI, O, S
11	1377	912	2,3-Dimethylpyrazine	Nut, peanut butter, cocoa, meat	32	RI, O, S
12	1389	942	4-Mercapto-4-methylpentan-2-one	Black currant	8	RI, O, S
13	1395	1020	2,3,5-Trimethyl pyrazine	Roast, potato, must	32	RI, O, S
14	1436	1006	2-Ethyl-3,6-dimethylpyrazine	Roast	> 64	RI, O, S
15	1442	1074	2-Ethyl-3,5(6)-dimethylpyrazine	Roast, potato	16	RI, O, S
16	1445	915	2,3-Diethylpyrazine	Baked	8	RI, O, S
17	1463	905	3-(Methylthio)propionaldehyde	Cooked potato	16	RI, O, S
18	1497	997	Benzaldehyde	Fruity, berry	8	RI, O, S
19	1647	863	furfurol	Burnt sugar	16	RI, O, S
20	1765	1286	2,4-Decadienal	Fatty, deep fried	32	RI, O, S
21	1822	981	Hexanoic acid	Sweaty, cheesy	16	RI, O, S
22	1955	1083	Heptanoic acid	Sweaty	8	RI, O, S
23	2163	1493	Decalactone	Coconut	4	RI, O, S

*RI* retention indices

^a^Methods of identification: *RI* retention index; *O* odor quality by GC–O; *S* chemical standard

With an FD factor of 32, 5 odor regions were detected, including dimethyl disulfide (cabbage/putrid), hexanal (fatty/apple/cut grass), 2,3-dimethylpyrazine (nut/peanut butter/cocoa), 2,3,5-trimethyl pyrazine (roast/potato) and 2,4-decadienal (fatty/deep fried), respectively. Odor threshold values of aldehydes are generally low thus they have important potential effect to the overall aroma of CCA. Hexanal was another influential aroma compound identified in CCA, which is widespread, as it has already been found in many other foods and fruits. 2,4-Decadienal is a powerful compound that contributes to the fatty note of CCA.

In addition, dimethyl sulfide (cooked onion/sulfur), 2,5-dimethylpyrazine (roasted nut/roast beef/medicine), 2-ethyl-3,5(6)-dimethyl pyrazine (roast/potato), 3-(methylthio) propionaldehyde (cooked potato), furfurol (burnt sugar), and hexanoic acid (sweaty, cheesy) were also suggested as key contributors to the overall aroma of CCA (Table 2). Dimethyl sulfide becomes highly disagreeable at even quite low concentration which has been characterized as the “smell of the sea”. Beetroot, asparagus, cabbage, corn and seafoods produce dimethyl sulfide when cooked and some researchers found it in red wine [30]. It will contribut to the muttony note of CCA.

### GC–MS/FPD Results

The volatile compounds identified in CCAs by SDE combined with GC–MS/FPD were listed in Table 3. A total of 65 compounds were identified and quantified in the 19 CCAs, including aldehydes, pyrazines, alcohols, ketones, esters, terpenes, lactones, carboxylic acids, furans, phenols and sulfur compounds. The highest amount of volatiles was found in S14 CCA (16610.4 μg/kg), followed by S5 (14973.2 μg/kg) and S6 (13046.2 μg/kg).

**Table 3:** Volatile compounds identified in The 19 CCA samples

No.	RI		Aroma compounds	Concentration^a^ (μg/kg)		Identification^b^
	INNOWAX	DB-5		Range	SD	
1	716	505	Dimethyl sulfided	0.1–0.8	0.01	RI, FPD, S
2	767	754	s-ethyl thioacetate	0–0.3	0.02	RI, FPD, S
3	1024	692	2-Ethyl-furan	0.3–893.9	26.3	RI, MS, S
4	1061	794	Dimethyl disulfide	0.1–0.5	0.02	RI, FPD, S
5	1075	701	s-methyl thioacetate	0.1–0.4	0.01	RI, FPD, S
6	1097	802	Hexanal	72.3–2073.5	78.3	RI, MS, S
7	1098	821	3-Methyl-2-butene-1-thiol	0–0.3	0.02	RI, FPD, S
8	1131	754	Pentanal	4.4–289.7	22.7	RI, MS, S
9	1174	901	Heptanal	1.9–308.5	10.5	RI, MS, S
10	1176	795	2-Methylpyrazine	0.9–1079.4	13.3	RI, MS, S
11	1238	989	2-Pentyl-furan	1.4–984.0	21.6	RI, MS, S
12	1243	844	(E)-2-hexenal	0.3–72.9	1.9	RI, MS, S
13	1244	967	2-Octanone	0.2–75.3	2.5	RI, MS, S
14	1245	905	Styrene	0.1–20.0	0.6	RI, MS, S
15	1247	956	(Z)-2-Heptenal	0.4–693.4	7.9	RI, MS, S
16	1280	992	Octanal	5.7–577.0	39.8	RI, MS, S
17	1311	961	(E)-2-heptenal	1.1–1383.9	15.4	RI, MS, S
18	1313	976	1-Octen-3-one	0.1–38.8	0.6	RI, MS, S
19	1320	979	2,3-Octanedione	0.1–152.1	1.8	RI, MS, S
20	1328	905	2,5-Dimethylpyrazine	19.9–6566.9	239.6	RI, MS, S
21	1330	885	2,6-Dimethylpyrazine	1.6–1209.9	31.1	RI, MS, S
22	1341	976	2-Ethyl-5-methylpyrazine	0.1–1919.9	39.7	RI, MS, S
23	1377	911	2,3-Dimethylpyrazine	0–378.8	7.4	RI, MS, S
24	1388	1075	2-Nonanone	1.4–1761.1	30.5	RI, MS, S
25	1389	942	4-Mercapto-4-methylpentan-2-one	0–0.2	0	RI, FPD, S
26	1395	1020	2,3,5-Trimethyl pyrazine	1.2–2003.6	45.3	RI, MS, S
27	1396	1075	Nonanal	7–1259.4	30	RI, MS, S
28	1412	1031	(3E)-3-ethyl-2-methyl-1,3-hexadiene	0.4–119.2	4.9	RI, MS tent
29	1432	1068	3,5-Octadien-2-one	0.1–48.3	0.6	RI, MS, S
30	1435	1034	2-Octenal	0.1–1091.7	32.2	RI, MS, S
31	1436	1006	2-Ethyl-3,6-dimethylpyrazine	0.3–822.1	18.4	RI, MS, S
32	1438	969	1-Octen-3-ol	0.2–149.0	6.6	RI, MS, S
33	1442	1074	2-Ethyl-3,5(6)-dimethylpyrazine	1.1–1735.2	20.3	RI, MS, S
34	1445	915	2,3-Diethylpyrazine	0.1–29.9	0.3	RI, MS, S
35	1449	625	Acetic acid	0.1–1083.2	15.7	RI, MS, S
36	1452	1074	3-Ethyl-2,6-dimethylpyrazine	0.1–389.2	4.2	RI, MS, S
37	1456	831	2-Furancarboxaldehyde	0.2–273.1	3.3	RI, MS, S
38	1459	1068	Tetramethyl-pyrazine	0.1–125.3	6	RI, MS, S
39	1463	905	3-(Methylthio)propionaldehyde	0.1–0.5	0	RI, FPD, S
40	1478	1171	2-Decanone	0.1–1.65.4	22.2	RI, MS, S
41	1501	996	Benzaldehyde	9.5–1872.5	64.4	RI, MS, S
42	1532	1135	2-Nonenal	0.2–1021.9	17.1	RI, MS, S
43	1539	1053	1-Octanol	0.2–303.3	8.8	RI, MS, S
44	1543	1276	2-Undecanone	0.1–10.5	0.4	RI, MS, S
45	1555	938	5-methyl furfural	0.1–31.4	0.9	RI, MS, S
46	1605	1110	2-Acetyl-3-methylpyrazine	0.5–151.1	2.3	RI, MS, S
47	1647	863	Furfurol	0.2–394.0	13.2	RI, MS, S
48	1655	825	3-Methylbutanoic acid	0.3–1042.3	9.4	RI, MS, S
49	1711	1169	2,4-Nonadienal	0.2–91.7	2.7	RI, MS, S
50	1724	938	Pentanoic acid	0.2–33.1	0.6	RI, MS, S
51	1741	1311	Azulene	0.4–27.6	0.3	RI, MS, S
52	1760	1339	2-Undecenal	0.4–443.4	25.1	RI, MS, S
53	1765	1286	2,4-Decadienal	0.1–570.6	8.1	RI, MS, S
54	1822	981	Hexanoic acid	0.6–105.4	4.5	RI, MS, S
55	1871	1286	2-Methyl-naphthalene	0.2–137.4	3.9	RI, MS, S
56	1899	1060	Phenethyl alcohol	0.1–195.8	3.3	RI, MS, S
57	1955	1083	Heptanoic acid	0.3–91.0	2.8	RI, MS, S
58	1970	1036	2-Acetyl pyrrole	0.1–49.8	1.2	RI, MS, S
59	1995	980	Phenol	12.9–107.2	5.7	RI, MS, S
60	2010	1679	2-Pentadecanone	0.1–85.8	2.1	RI, MS, S
61	2018	859	2(3H)-furanone, dihydro-	0.1–50.4	0.6	RI, MS, S
62	2084	1059	p-cresol	1.0–567.8	7.4	RI, MS, S
63	2148	1272	Nonanoic acid	0.3–125.0	6.2	RI, MS, S
64	2163	1493	Decalactone	0.1–56.5	0.8	RI, MS, S
65	2468	1554	Dodecanoic acid	0.1–326.7	4.3	RI, MS, S

*RI* retention indices

^a^Results are mean of three replications; *SD* standard deviation

^b^Methods of identification: *RI* retention index; *MS tent* tentatively identifided by MS; *FPD* flame photometric detector; *S* chemical standard

Among the 65 identified volatiles, aldehydes are the most abundant chemical group found in CCA. A total of 13 aldehydes were identified in all CCAs. The S1 (5836.6 μg/kg), S15 (5554.6 μg/kg) and S19 (3676.3 μg/kg) CCAs were characterized by the highest aldehyde contents. Aldehydes are generally characterized by intense sensory description by panelists associated with green, cut grass, citrusy and sweet notes. Hexanal, nonanal and benzaldehyde were the major components among aldehydes. (E)-2-hexenal has been demonstrated to be a predominant compound in guava [31]. These unsaturated aldehydes are generally considered as the oxidized products of unsaturated fatty acids.

Pyrazine compounds were the second largest class of volatile compounds in CCA samples. A total of 12 aldehydes were identified in all CCAs, including 2,5-dimethylpyrazine, 2-ethyl-5-methylpyrazine, 2-ethyl-3,5(6)-dimethylpyrazine, and so on (Table 3). The total concentration of pyrazine compounds in the S14 CCA (13255.9 μg/kg) was overwhelmingly higher than in other CCA samples.

Eight ketones were found in the CCAs, including 2-octanone, 1-octen-3-one, and 2,3-octanedione. They contribute to the milky, waxy, earthy and fatty aroma. The highest concentration of ketones was found in S5 CCA (2597.3 μg/kg) and the lowest amount of ketone compounds was found in S11 CCA (7.5 μg/kg). Significant differences were observed in terms of the contents of ketones compounds.

Volatile sulfur compounds are typically in the parts per trillion (ppt) range to low parts per billion (ppb) range [32], however, they are normal components of wine, beer, cheese or cooked meat aroma, and can even exert a decisive role on some characteristic sensory descriptors [33]. As shown in Table 3, a total of 7 sulfur volatiles were detected in the 19 CCAs. Some aroma-active sulfur volatiles, including dimethyl sulfide, dimethyl disulfide, s-methyl thioacetate, ethanethioic acid s-ethyl ester, 3-methyl-2-butene-1-thiol, 4-mercapto-4-methylpentan-2-one and 3-(methylthio) propionaldehyde, have very strong aroma notes, which were described as possessing “cooked onion, sulfuric”, “cabbage-like, rotten egg”, “sulfurous, burnt”, “sulfurous”, “amine, smoke”, “black currant” and “cooked potato” aromas, respectively. The primary sources for the sulfur-containing volatiles are the degradation of the sulfur-containing amino acids, methionine and cysteine, which have been found in CCA [34]. The relative levels of these amino acids in different CCA samples are probably responsible for the differences in the sulfur volatiles. Among the sulfur volatiles, four compounds, namely dimethyl sulfided, dimethyl disulfide, s-methyl thioacetate and 3-(methylthio) propionaldehyde, were observed in all the 19 CCA samples. The highest amount of sulfur compounds was found in S5 CCA (3.0 μg/kg), followed by S6 (2.6 μg/kg) and S19 (2.6 μg/kg). Sulfur compounds had been previously reported in CCA and they are extremely potent and produce only weak sulfur FPD peaks. At the normal attenuation their peaks would generally be overlooked and no further examination would be performed without GC–O analysis indicating strong aroma signals at the corresponding retention times. Therefore, GC–O is a useful complementary tool for analysis of some extremely low-content and potent aroma components.

### Principal Components Analysis (PCA) and Correlation Analysis

In order to study the potential application of aroma analysis to the quality evaluation and discrimination of different CCAs, the contents of the 23 aroma-active components in the 19 CCAs were submitted to PCA. Considering the fact that the odor level of each aroma-active component depends on both its content and threshold, the contents of aroma-active components in the 19 CCAs were mean centered and scaled to have a unit sample standard deviation. The first three principal components (PCs) jointly explained 71.80% of the total data variance observed. There were at least 5 very significant PCs, which accounted for 43.35, 17.25, 11.20, 6.44 and 6.26% of the total variance, respectively. Moreover, 7 (92.46%) and 9 PCs (96.97%) were required to explain 90 and 95% of the total data variances, respectively. The large number of significant PCs indicate the factors influencing the aroma of CCAs from different producing areas were multiple and complicated.

**Fig. 1:**
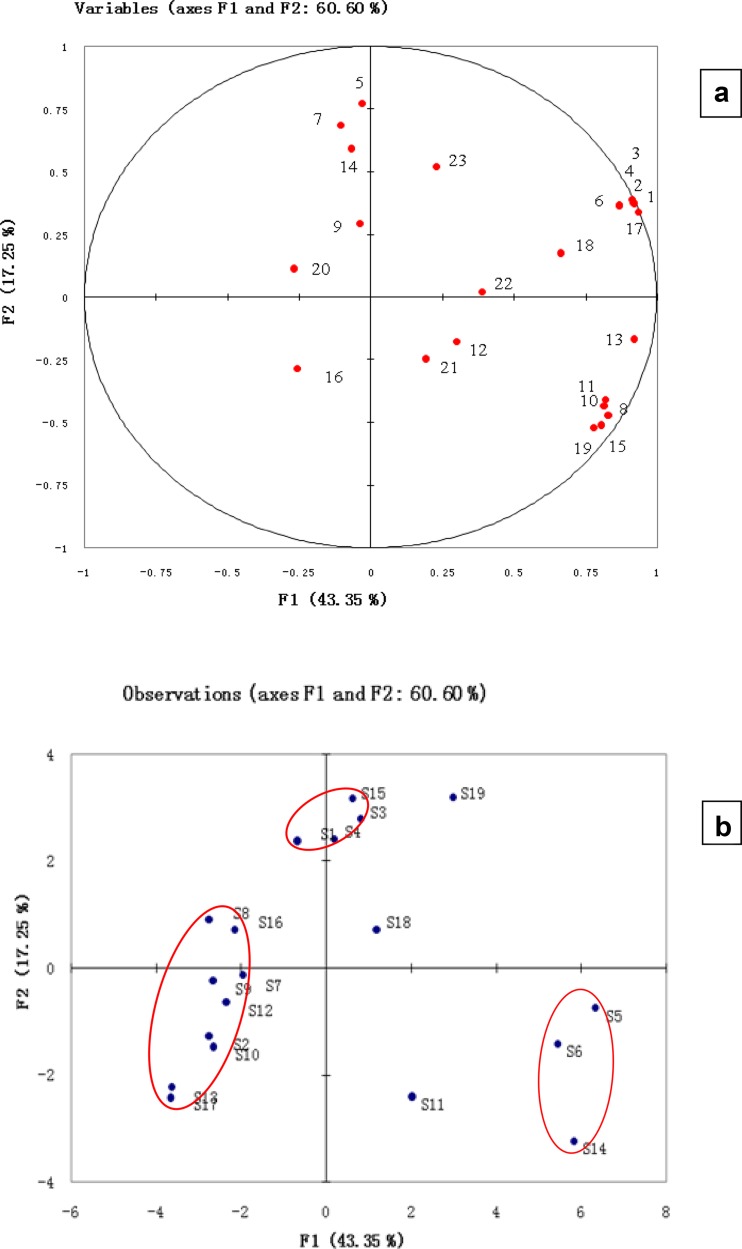
PCA loading plots and score for 19 CCAs. **a** Loading plot; **b** score plot

Figure 1a represents the corresponding loadings plot that established the relative importance of each odour-active compound and the relations between odour-active compounds and samples. Main volatiles positively correlated to PC1 were 3-(methylthio) propionaldehyde (17), 2,3,5-trimethyl pyrazine (13), dimethyl sulfided (1) and dimethyl disulfide (3). According to the loading plot, hexanal (5), 2-pentyl-furan (7), 2-ethyl-3,6-dimethylpyrazine (14) were positively correlated to PC2. Combined with Fig. 1b, it is obvious that hexanal (5), 2-pentyl-furan (7), 2-ethyl-3,6-dimethylpyrazine (14) were important to the aroma of Dong-E CCA and the results are similar to those by AEDA assay.

Figure 1b shows the scores scatter plot on PC1 and PC2. The score plot indicates that the CCA samples are not arranged in a random way but could be generally divided into three groups (Fig. 1b). It is obvious that the levels of odour-active compounds in CCAs from different regions are distinctively different. In this study, we found that the chemical character of the samples from the HeNan (S11), BeiJing (S18) and GanSu (S19) provinces of China was significantly different from any of the three groups owing to the unique geographical factors.

The squares of correlation coefficients (r^2^) among the 19 CCAs were computed to investigate their similarity based on the 23 aroma-active components. The r^2^ of each pair among the 19 CCAs was less than 0.8 and the two highest r^2^ values were 0.7132 (S16 and S7) and 0.6018 (S14 and S6). The low similarity in terms of correlation coefficients among the 19 CCAs indicates that the contents of the 23 aroma-active components are specific to the producing areas of CCAs. Therefore, the profiles of aroma volatiles demonstrate the feasibility and potential for discriminating the producing areas of CCAs.

### Sensory Assessment of CCAs

**Fig. 2:**
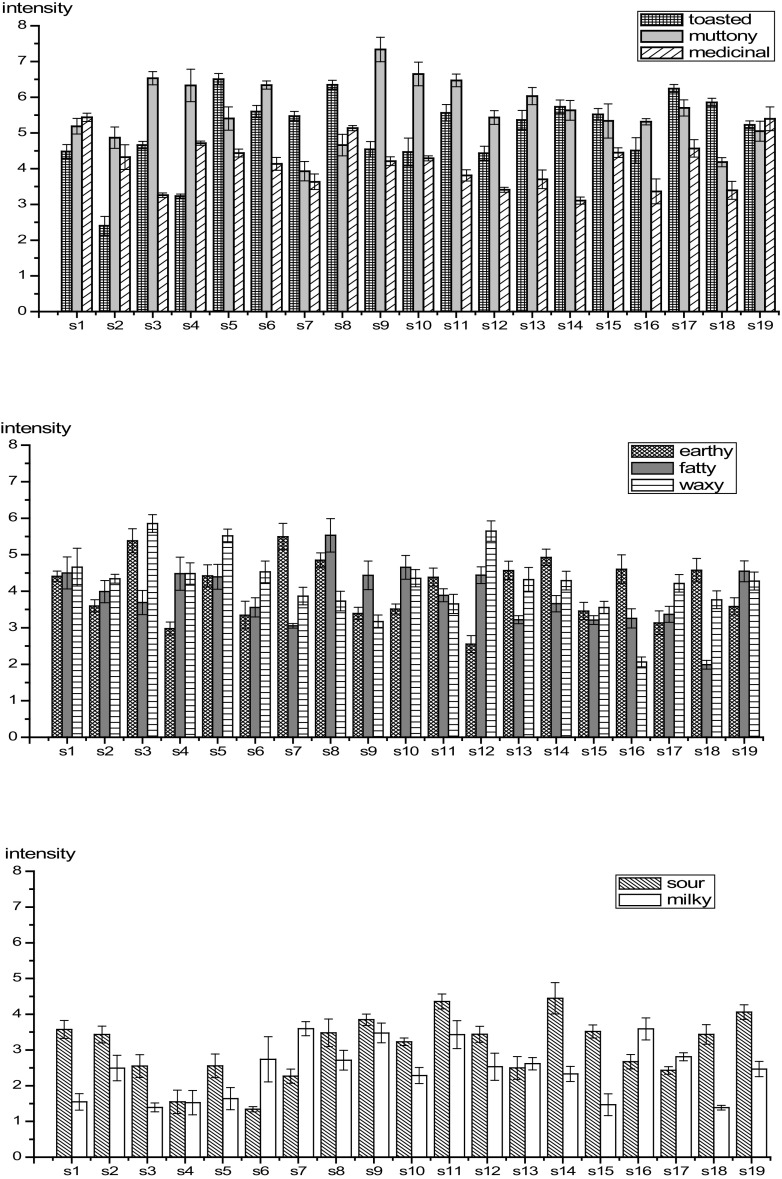
Sensory properties of the 19 CCAs

Figure 2 is a summary of the total aroma grouped into eight regions (toasted, muttony, medicinal, fatty, milky, earthy, sour and waxy) based on aroma similarities by sensory analysis. It can be seen from Fig. 2 that the toasted, muttony and medicinal note characters are the major contributors to CCA aroma. The fatty, waxy and earthy notes are midlevel aroma attributes. Finally, the remaining two aroma categories, sour and waxy contribute to background CCA aroma. According to sensory analysis, the relative aroma strengths of the 8 aroma categories varied somewhat between the 19 CCA samples. The strongest toasted aroma was found in S17 (6.25) and S2 (2.40) demonstrated the lowest toasted aroma. Sulfur compounds did play a key role in muttony note and S9 (7.84) had the highest value and S7 (3.93) had the lowest value. As to the medicinal note, S19 (5.40) possessed the strongest aroma and S14 (3.11) is the weakest. S7 (5.50), S8 (5.53) and S3 (5.85) exhibited highest in the earthy, fatty and waxy aroma categories, respectively.

The r^2^ among the 19 CCAs were also computed based on the sensory characters of CCAs. The r^2^ of each pair among the 19 CCAs was less than 0.8 and the two highest r^2^ values were 0.7132 (S16 and S7) and 0.6018 (S14 and S6). The conclusion from sensory analysis were generally consistent with that of aroma-active components analysis, indicating aroma is an important quality parameter of CCAs and its attributes largely depend on the producing areas of CCAs. Because sensory analysis is subjective and is less stable compared with GC–MS and GC–O, the latter two techniques will provide a useful alternative tool for quality evaluation and discrimination of geographical origins for CCA.

### Antioxidant Activities and Canonical Correlation Analysis

In the present study, the ability of test samples to scavenge ABTS is assessed on the bases of their IC_50_ values, defined as an effective concentration at which the ABTS radical was scavenged by 50%. IC_50_ values of CCAs S1–S19 were given in Table 4. A low IC_50_ value indicates strong antioxidant activity in a tested sample. In this assay, the most potent radical scavenger was S19 (2.094), followed by S6, S18, S2, S17, S14, S15, S3, S4, S16, S1, S13, S12, S5, S10, S11, S9, S8 and finally S7(4.778).

**Table 4:** Antioxidant capacity by ABTS and DPPH radical scavenging assays

Samples	Antioxidant capacity^a^	
	ABTS radical scavenging assay	DPPH radical scavenging assay
	(IC_50_ ± SD, g/L)	(IC_50_ ± SD, g/L)
S1	2.859 ± 0.476	24.974 ± 1.168
S2	2.442 ± 0.295	22.893 ± 2.457
S3	2.734 ± 0.291	24.810 ± 3.821
S4	2.741 ± 0.289	24.878 ± 2.916
S5	3.426 ± 0.043	26.267 ± 3.223
S6	2.104 ± 0.237	23.977 ± 5.528
S7	4.778 ± 0.251	33.698 ± 5.287
S8	4.571 ± 0.050	30.718 ± 2.550
S9	4.168 ± 0.137	30.147 ± 8.397
S10	3.578 ± 0.370	27.202 ± 3.847
S11	3.745 ± 0.110	28.319 ± 4.538
S12	3.360 ± 0.208	26.087 ± 3.246
S13	3.202 ± 0.429	25.703 ± 4.140
S14	2.708 ± 0.187	24.637 ± 5.057
S15	2.710 ± 0.254	24.841 ± 3.371
S16	2.802 ± 0.103	21.356 ± 2.652
S17	2.520 ± 0.150	25.944 ± 2.654
S18	2.390 ± 0.082	21.565 ± 1.748
S19	2.094 ± 0.190	18.827 ± 1.706

^a^All values are mean ± SD of three replications with a 95% confidence interval

The ability of test samples to scavenge DPPH is assessed on the bases of their IC_50_ too [35]. The effect of antioxidants on DPPH radical scavenging is generally due to their hydrogen-donating ability. The reduction capacity of DPPH radicals was determined by the decrease in its absorbance at 517 nm [36]. The 19 CCA samples differed in their DPPH radical cation scavenging capacities (Table 4). S19 (18.827) showed higher antioxidant capacity than other samples in terms of DPPH. The results were similar to that of ABTS assays. In brief, the DPPH radical cation scavenging capacities of the nineteen samples exhibited the descending order of S19, S16, S18, S2, S6, S14, S3, S15, S4, S1, S13, S17, S12, S5, S10, S11, S 9, S8 and S7.

The correlation coefficient between DPPH and ABTS assays was 0.9117, indicating the extraction of CCAs were well controlled and antioxidant activities analysis was reliable. To further investigate the relationship between aroma-active components and the antioxidant activities, canonical correlation analysis was performed between the antioxidant activities (DPPH and ABTS assay values) and the contents of 23 aroma volatiles. Considering the number of aroma-volatile variables (23) is much larger than that of antioxidant activities (2), only the first 9 PCs of aroma volatiles (explaining over 95% of the total variance) instead of the 23 raw variables were used in canonical correlation analysis to avoid overfitting. The largest canonical correlation coefficients were 0.9776 (p = 0.0281). The results of canonical correlation analysis indicated that the antioxidant activities of CCAs were highly correlated with the aroma volatiles and the latter could reflect the quality of CCAs.

## Conclusion

In the present study, 65 major volatile compounds were identified in CCA by SDE/GC–MS/FPD analysis, and 23 potent odorants have been identified by GC–O analysis. Through AEDA the main aroma-active compounds identified as being responsible for the typical aroma in CCA were s-methyl thioacetate, 2,6-dimethylpyrazine and 2-ethyl-3,6-dimethylpyrazine.

Moreover, PCA was performed to show the distribution of CCA samples and the relationship between aroma-active compounds and CCA samples. According to the loading plot, hexanal, 2-pentyl-furan and 2-ethyl-3,6-dimethylpyrazine were representative aroma compounds of Dong-E CCA. The result is similar to that obtained by aroma extract dilution analysis (AEDA).

Finally, the antioxidant activities of 19 kinds of CCA were evaluated by DPPH radical scavenging activity and ABTS radical cation scavenging activity. Canonical correlation analysis has proved the relationship between antioxidant activities and the 23 aroma-active components.

Analysis of aroma-active components shows potential for quality evaluation and discriminaton of CCAs from different geographical origins. Future investigations will be focused on the influencing factors of CCA antioxidant activities.
